# Proprioceptive elbow training reduces pain and improves function in painful lateral epicondylitis—a prospective trial

**DOI:** 10.1186/s13018-021-02602-3

**Published:** 2021-07-27

**Authors:** B. Schiffke-Juhász, K. Knobloch, P. M. Vogt, L. Hoy

**Affiliations:** 1 Herzogin Elisabeth Hospital, Braunschweig, Germany; 2 Sportpraxis Knobloch, Hannover, Germany; 4 Medizinische Hochschule Hannover, Hannover, Germany; 3 Formerly Medizinische Hochschule Hannover, Hannover, Germany

**Keywords:** Epicondylitis humeri radialis, Tennis elbow, Proprioception, Proprioceptive exercise

## Abstract

**Background:**

In painful epicondylitis, previous studies reported deficiencies in elbow proprioception. In line, proprioceptive training of the lower limb has been reported substantial beneficial in a number of indications. Therefore, we have asked if a specified proprioceptive training using training devices that are capable of activating the deep musculature in the upper limb is able to reduce the symptoms of epicondylitis.

**Materials and methods:**

We included 71 patients with painful lateral epicondylitis > 3 months. Interventions: Group A: Proprioceptive training intervention with a Flexibar® (9 min daily for 12 weeks). Group B: at least 40 min running or walking/week with the XCO® in addition to the proprioceptive training with the Flexibar® (9 min daily for 12 weeks), follow-up for 12 weeks. Primary end point: Pain on visual analogue scale (VAS, 0-10); secondary end points: DASH-Score (0 = very good, 100 = very poor), grip strength according to Jamar dynamometer (kg), vibration sensation measured with a 128 Hz tuning fork.

**Results:**

The pain on VAS in group A was reduced significantly. 3.6 ± 2.0 to 2.4 ± 2.1 (−33%, *p* = 0.013), and from 3.7 ± 2.4 to 2.2 ± 1.9 (−41%, *p* = 0.004) in group B after 12 weeks. There was no significant difference between A and B (*p* = 0.899). In both groups, there was a significant improvement of the DASH-Score (A: 32 ± 15 to 14 ± 12, −56%, *p* < 0.001; B: 27 ± 12 to 12 ± 11, −55%, *p* = 0.001) without any difference between groups A and B (*p* = 0.339). Grip strength improvement in group A from 24 ± 12 to 33 ± 11 kg (+38%, *p* < 0.001), and from 29 ± 14 to 34 ± 11 kg (+15%, *p* < 0.001) in group B. In line, vibration sensation improved in both groups (A: 6.3 ± 0.6 to 6.5 ± 0.5, *p* = 0.0001; B: 6.3 ± 0.7 to 6.6 ± 0.5, *p* = 0.003).

**Conclusion:**

A 12-week proprioceptive training with the Flexibar® improves pain, quality of life, grip strength and vibration sensation in patients with painful lateral epicondylitis.

**Level of evidence:**

Ib, randomised clinical trial

**Trial registration:**

German Clinical Trials Register, DRKS00024857, registered on 25 March 2021—retrospectively registered, http://apps.who.int/trialsearch/

## Background

The epicondylitis humeri radialis, often simply referred to as tennis elbow, appears with an incidence of 1-3% in the population [1, 2], and occurs most frequently between the third and fifth decade of life [1]. Amongst women between the 40th and 50th year of life the disease may occur with a prevalence of 10% [2]. Thereby, the epicondylitis humeri radialis is not only a very widespread disease but also affects more often the working population. Even though the aetiology of the disease has still not been fully identified and it seems to be determined multifactorially, a number of studies have shown that there are various risk factors, for example, repetitive movements, forceful work or activities which require an unnatural posture of hands and arms [3, 4]. Such stresses cannot only be found amongst active tennis players but also within a lot of professional activities, so that only a small part of the patients with lateral epicondylitis exercises the name-giving sport at all [1]. Another risk factor relating to a low social support at work could be identified amongst female patients [3]. Furthermore, there seems to be a relation with a current or previous nicotine abuse [3, 4]. In addition, further studies were able to demonstrate reduced deficiencies in proprioception in patients with symptomatic lateral epicondylitis [5].

Even though Cyriax already described, in 1936, a spontaneous consolidation of the symptoms after about 8-12 months on the condition of functional physical rest [1], most patients expect a quicker therapeutical intervention, so that the question concerning an appropriate therapy is further relevant. At this point, a very wide spectrum of therapeutical options is available for therapists, which were repetitively compared against each other within various studies. In this context, it could be shown that there is no universal solution for the therapy of the lateral epicondylitis, but that patients benefit from various therapeutic approaches. Whereas surgical treatment only play a role in cases of failure of the conservative therapeutic options [1, 6], there are, besides rarely applied conservative therapeutic options, often treatments using orthoses, injections and physiotherapy in the foreground. In case of a treatment using orthoses, it can be chosen from a lot of different types of orthoses, whereby a number of studies could not prove relevant differences in the outcome of the different types of orthoses [7], though Garg et al. could indeed find a slight advantage in the pain reduction of using wrist extension splints rather than using usual epicondylitis braces [8]. However, the pain relief is here tied to wearing the orthosis, whereby the affected muscles only get a relief but no sustainable functional optimisation. Another frequently used therapeutic option is a local injection, most common with a medical preparation of cortisone (rarer used are PRP, Botox, or others). In doing so, it is essential to take into account not only the therapeutic effect but also the risk potential due to systemic complications (for example multifocal osteonecrosis or other well-known systemic side effects) and the possibility of local complications (for example tendon or fascial ruptures) [9]. As demonstrated in various studies, the therapeutic effect of injection treatment seems to be only superior in the short-term follow-up to the physiotherapeutic options [10]. With regard to the use of PRP, Ang Li et al. found inferiority to corticosteroids after 4-8 weeks, but superiority of PRP in the long term (after 24 weeks) [11]. Ruiz et al. were also able to show improvements in symptoms for the use of botulinum toxin, but accompanied by a certain weakness of the 3rd finger, without other side effects [12]. Another study by Newcomer et al. ultimately identified rehabilitation as the first-line therapy for short-lasting symptoms [13]. However, it should be noted that the repertoire of physiotherapeutic options with over 40 different methods [6] is extremely wide spread, so that even at this point a calculated decision regarding a suitable therapeutic option becomes necessary. As deficiencies in proprioception can play a role in different tendon diseases which was explicitly proven by Juul-Kristensen et al. for patients with lateral epicondylitis compared to healthy control subjects [5], we aspired to design a training concept to improve the proprioceptive capabilities regarding the affected area. In this context, we have chosen to examine not only the training with one or two appropriate training devices to run a prospective, randomised clinical trial with regard to the improvement of the proprioceptive capabilities but also the clinical development of the symptoms.

### Hypothesis

The combination of a proprioceptive and ballistic training is effective for patients with lateral epicondylitis regarding pain reduction and functional improvement.

## Materials and methods

Agreement of the ethics committee: Medizinische Hochschule Hannover, Germany.

### Inclusion criteria

Patients suffering epicondylitis humeri radialis for at least 3 months, verified by clinical examination with exercise related pain, at the age of 18 to 65 years. Therefore used diagnosis criteria were pain on palpation of the lateral epicondyle as well as the declaration of pain during provocation tests.

### Exclusion criteria

Epicondylitis humeri ulnaris, missing consent, beginning of different therapeutic treatments during the study phase.

### Patient characteristics

**Fig. 1:**
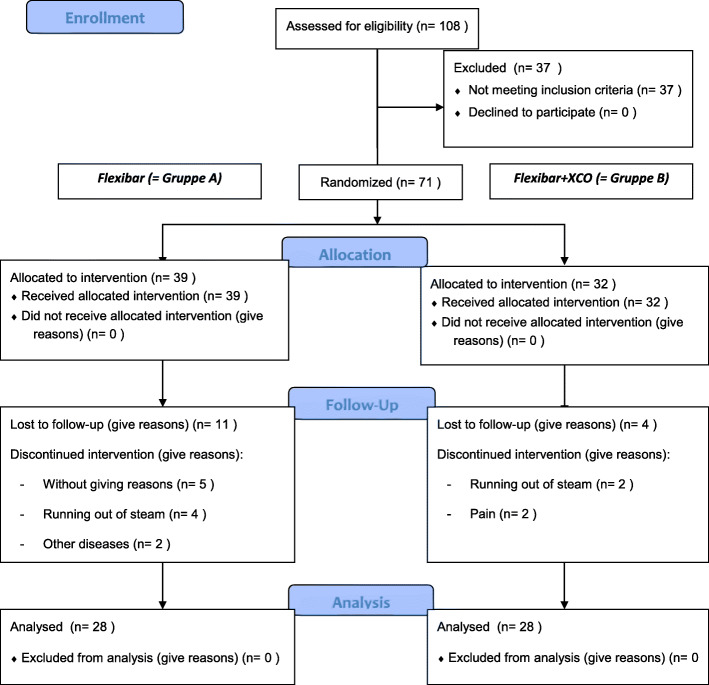
Flow chart of the study protocol

Figure 1 illustrates the course of the study in form of a CONSORT flow chart. This includes an evaluation of 108 patients to verify clinically the existence of a lateral epicondylitis by medical history as well as performing significant clinical tests (pain on palpation of the lateral epicondyle, painful extension of fingers against resistance). Seventy-one patients with lateral epicondylitis were subsequently randomised by lottery into two groups. The lottery has taken place by drawing an opaque lot with the designation ‘Group A’ or ‘Group B’. The patients’ characteristics of both groups are shown in an overview in Tables 1 and 2.

**Table 1:** General characteristics

	Group A (Flexibar®)	Group B (Flexibar®+XCO®)	Chi-square test
Basic characteristics			
Gender	*m* = 15; *f* = 24	*m* = 17; *f* = 15	0.217
Age [years]	47 ± 8	47 ± 8	0.173
Weight [kg]	72 ± 15	76 ± 12	0.300
Height [cm]	173 ± 8	175 ± 10	0.006
BMI	24.2 ± 4.3	24.7 ± 3.5	0.352
Underlying diseases			
Nicotine abuse	15%	13%	0.728
Diabetes	3%	0%	0.362
Hypercholesterolemia	8%	9%	0.800
Hypertension	8%	13%	0.499
Heart disease	0%	3%	0.266
Intake of acetylsalicylic acid	5%	6%	0.838
Intake of cortisone (spray)	3%	13%	0.103
Diseases of the elbow			
Family disposition	neg. = 29; pos. = 10	neg. = 27; pos. = 5	0.341
Pain of right elbow	74%	69%	0.539
Pain of left elbow	5%	13%	0.539
Bilateral pain of elbow	21%	19%	0.539
Morning stiffness	33%	28%	0.585
Swelling	15%	13%	0.695
Pressure pain	90%	88%	0.522
Duration of pain	> 27 weeks	> 27 weeks	0.485
Pain in the morning [VAS]	2.7 ± 2.2	2.5 ± 1.8	0.465
Daily maximum of pain [VAS]	5.2 ± 1.8	4.7 ± 2.2	0.076
Intake of antibiotics (Ciprobay, Tavanic)	3%	0%	0.362
Previous therapies			
Massage	51%	41%	0.424
Cross friction	38%	16%	0.060
Heat application	18%	22%	0.102
Cold application	44%	38%	0.371
Shock wave	15%	13%	0.111
Eccentric training	8%	0%	0.066
Sclerotherapy	0%	3%	0.126
Injection of corticosteroids	56%	63%	0.475
Injection of Traumeel	10%	0%	0.025
Surgical treatment	3%	6%	0.311
Bandage	79%	72%	0.169
Taping	10%	16%	0.176
Kinesiology taping	10%	9%	0.050
Collateral tendon diseases			0.369
Pain of the patella tendon	5%	9%	
Pain of the Achilles tendon	0%	6%	
Sulcus ulnaris syndrome	3%	3%

**Table 2:** Sports and profession

	Group A (Flexibar®)	Group B (Flexibar®+XCO®)	Chi-square test
Sports			
Constant sporting activity	80%	91%	0.369
Weekly training sessions	1.6 ± 0.7 à 1.7 ± 1.3 h	1.5 ± 0.6 à 1.6 ± 0.7 h	0.226
Professional groups			0.271
Employee	54%	56%	
Engineer	5%	13%	
Independent	3%	3%	
Housewife	5%	0%	
Sports teacher/therapist	0%	6%	
Pensioner	5%	0%	
Civil servant	18%	9 %	
Craftsman	5%	6%	
Lawyer	0%	6%	
Physician	3%	0%	
Use of PC			
Professional use of PC	87%	91%	0.648
Hours of professional use of PC per day	4.9 ± 2.6	5.3 ± 2.7	0.545
Private use of PC	92%	88%	0.768
Hours of private use of PC per day	0.9 ± 0.5	1.0 ± 0.9	0.221

### Intervention

**Fig. 2:**
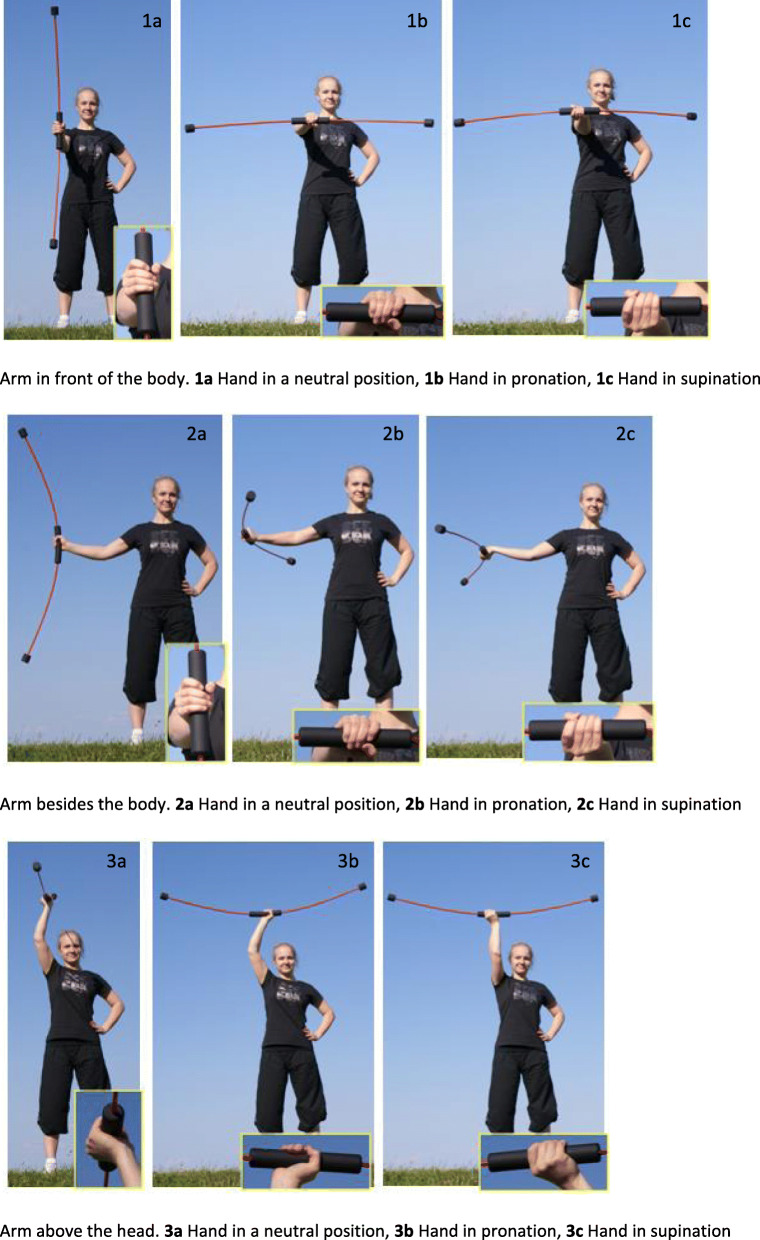
Instructions for the training. Arm in front of the body: **1a** Hand in a neutral position, **1b** hand in pronation, **1c** hand in supination. Arm besides the body: **2a** Hand in a neutral position, **2b** hand in pronation, **2c** hand in supination. Arm above the head: **3a** Hand in a neutral position, **3b** hand in pronation, **3c** hand in supination

The intervention in ‘Group A’ consisted in an at least 12-week training with the Flexibar®. The Flexibar® is a swing bar with rubber weights at the ends. The device is actively vibrated. The alternating vibration causes an additional reflexive tensioning of the deep muscles. We requested a daily training of at least 9 min, whereby the exercises shown in Fig. 2 ought to be performed in each case for 30 s per limb.

**Fig. 3:**
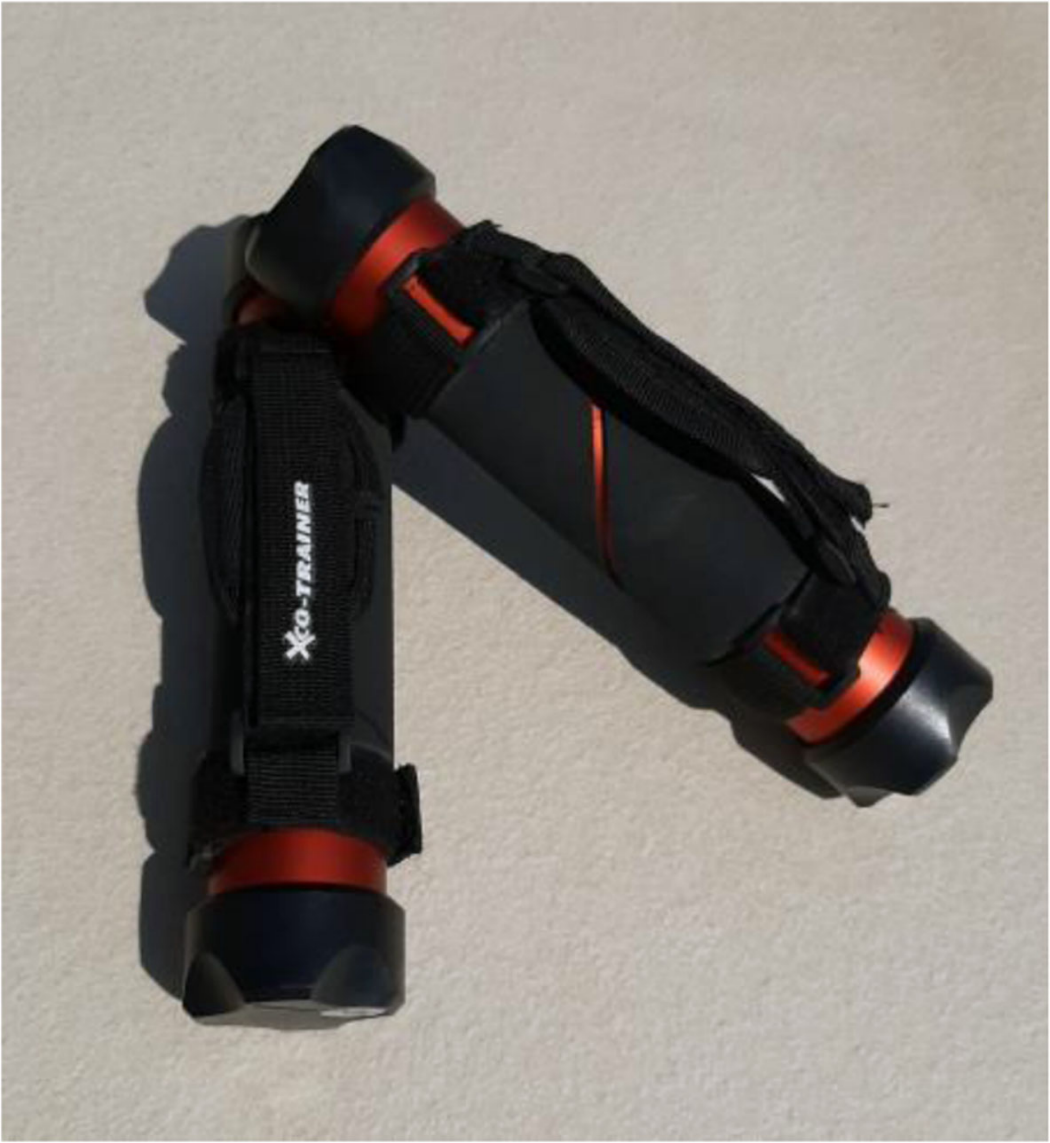
XCO® Walking & Running

Test subjects in ‘Group B’ were requested to do the same training programme, but received an additional other training device—the XCO® Walking & Running. The XCO® Walking & Running is a metal tube filled with a granulate (Fig. 3). The granulate is set in motion by the swing of the arm whilst walking and each time the granulate hits the ends of the tube, a so-called ‘reactive impact’ occurs, which in turn should lead to a reflexive, stabilising tensioning of the deep muscles. With that training device, the patients had to complete 2 further training sessions per week. Each training session should include at least 20 min of walking or running with the training device. Thereby, the XCO®-tubes should be held one in each hand and be moved with a powerful movement of the arms to achieve the effect described.

Patients of both groups were asked to keep a diary concerning their pain levels and also training sessions during the whole period of the study.

Before the start of the training phase and after terminating the 12 weeks period, we performed each time an examination, whereby different measurement parameters were collected and the patients had to fill in the DASH-Score (score for the disabilities of the arm, shoulder and hand). We measured the strength development according to Jamar, the 2-point discrimination at the distal phalanx of each finger, both radial and ulnar, the vibration sensation (by 128 Hz tuning fork) on top of the acromion, at the epicondylus humeri radialis and ulnaris, as well as at the distal ending of the radius and the ulna.

### Primary goal parameter

- Course of pain on the visual analogue scale (VAS 0-10)

### Secondary goal parameter

- DASH-Score (from 0 = no limitations to 100 = strong limitations)
- Compliance whilst performing the training period
- Measuring of the strength development according to Jamar [kg]
- Measuring the vibration sensation in 1/8 steps by using a 128 Hz tuning fork
- Measuring the 2-point discrimination at the distal phalanx of each finger both radial and ulnar

### Statistics

The input and processing of the statistical data was performed with SPSS 17.0. The patients’ characteristics were processed using cross tables and chi-square tests. For the analysis of the measured values, we initially calculated averages: average of the 3 attempts of the strength development, average of the 2-point discrmination for each hand, and average of the vibration sensation at the different localisations, etc. We subsequently calculated the changes of the paired *t* test, comparing before and after. For the comparison between both groups, we used the unpaired *t* test.

## Results

Randomisation of the patients to ‘Group A’ (Flexibar®) and ‘Group B’ (Flexibar® + XCO®) resulted in a distribution without relevant differences between the groups. The detailed list of basic characteristics, underlying diseases, diseases of the elbow and collateral tendon diseases can be found in Table 1. The table also shows that the patients in both groups reported symptoms for more than 27 weeks on average when they entered the study. In addition, the treatments already received in advance can also be taken from here. In addition, Table 2 shows the sporting activity, professional activity and PC use.

### Primary goal parameter

#### Course of pain on the visual analogue scale (VAS 0-10)

**Fig. 4:**
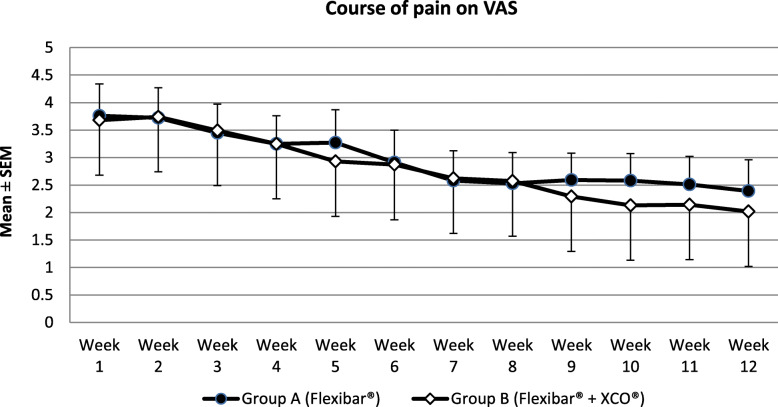
Course of pain on VAS

In ‘Group A’ (Flexibar®), there was a reduction of pain from 3.6 ± 2.0 to 2.4 ± 2.1, which is corresponding with a decrease of 1.2 ± 2.5 points on the pain scale and a *p* value of *p* = 0.013. In ‘Group B’ (Flexibar® + XCO®), we found a reduction of pain from 3.7 ± 2.4 to 2.2 ± 1.9 and therefore a decrease of 1.5 ± 2.4 points on the pain scale and a *p* value of *p* = 0.004. See also Fig. 4. There was no significant difference between ‘Group A’ (Flexibar®) and ‘Group B’ (Flexibar® + XCO®) (*p* = 0.899).

### Secondary goal parameter

#### DASH-Score (from 0 = no limitations to 100 = strong limitations)

**Fig. 5:**
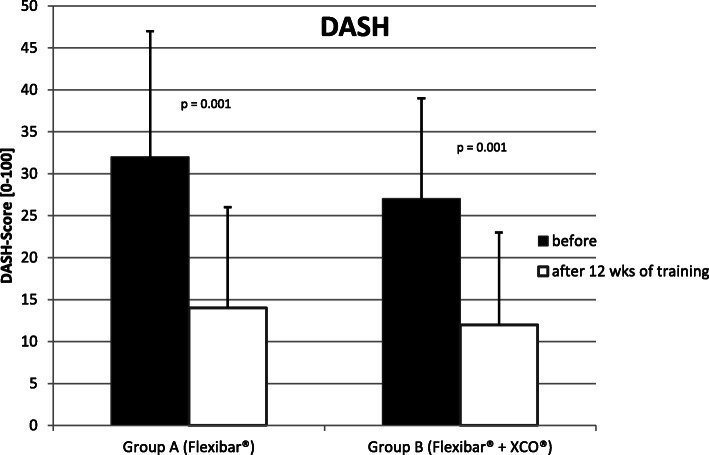
DASH-Score before and after the 12 weeks of training

Concerning the DASH-Score, an improvement was achieved from 32 ± 15 points before the period of training to 14 ± 12 points after the training phase in ‘Group A’ (Flexibar®). In ‘Group B’ (Flexibar® + XCO®), there was an improvement from 27 ± 12 points to 12 ± 11 points. This corresponds with an improvement of 44% in ‘Group A’ (Flexibar®) and 44 % in ‘Group B’ (Flexibar® + XCO®). We were thus able to observe a significant improvement relating to the symptomatic limb with a *p* value of *p* = 0.001 in both groups. This is corresponding with a change for the better of 56% in both groups (Fig. 5) and without significant difference between the groups (*p* = 0.677).

#### Compliance whilst performing the training period

The patients in ‘Group A’ (Flexibar®) trained with the Flexibar® on average over a period of 91 ± 12 days for 798 ± 223 min (corresponding on average to 106% of the required training), which complies with a weekly training of 61 ± 17 min and a daily training of 9 ± 3 min. On average, the patients in this group missed 12 ± 11 training sessions (corresponding on average to 15 % of the required training sessions).

**Fig. 6:**
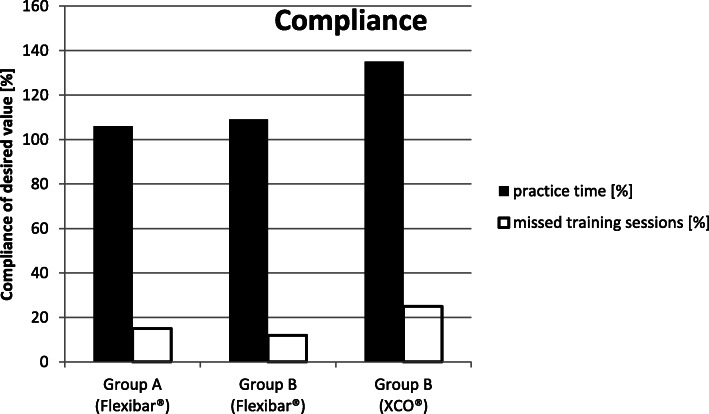
Synopsis of the compliance of the patients

The patients in ‘Group B’ (Flexibar® + XCO®) trained with the Flexibar® on average over a period of 95 ± 17 days for 825 ± 192 min (corresponding on average to 109% of the required training), which complies with a weekly training of 61 ± 14 min and a daily training of 9 ± 2 min. On average, the patients in this group missed 10 ± 10 training sessions (corresponding on average to 12% of the required training sessions). In the same period, they trained with the XCO® on average 648 ± 446 min (corresponding on average to 135% of the required practice time), which complies with a weekly training of 47 ± 27 min and 27 ± 12 min per training session. This practice time was spread on average over 2 ± 1 training sessions per week. In doing so, the patients in ‘Group B’ missed 6 ± 9 training sessions with the XCO® (corresponding on average to 25 % of the required training sessions) (Fig. 6).

#### Measuring of the strength development according to Jamar [kg]

The measurements were taken concerning the symptomatic limb by using the 3-attempt-method, each time with extended as well as with a flexed elbow.

In ‘Group A’ (Flexibar®), we found an improvement of the strength development concerning the symptomatic limb when testing with an extended elbow from 24 ± 12 kg to 33 ± 11 kg and when testing with a 90° flexed elbow from 26 ± 12 kg to 32 ± 9 kg. This is corresponding with an increase of the strength development of 38% (extended) or 23% (flexed). Therefore, in ‘Group A’ (Flexibar®), a significant improvement was achieved in both test positions with a *p* value of *p* = 0.001 (extended) and *p* = 0.004 (flexed).

**Fig. 7:**
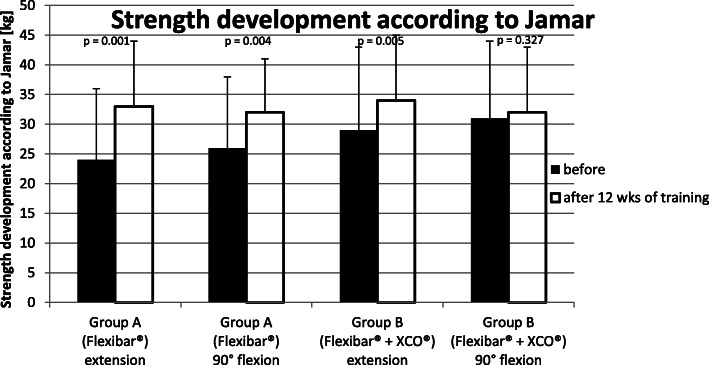
Strength development according to Jamar

In ‘Group B’ (Flexibar® + XCO®), we found an improvement of the strength development concerning the symptomatic limb when testing with an extended elbow from 29 ± 14 kg to 34 ± 11 kg and when testing with a 90° flexed elbow from 31 ± 13 kg to 32 ± 11 kg. This is corresponding with an increase of the strength development of 17% (extended) or 3% (flexed). Therefore, in ‘Group B’ (Flexibar® + XCO®), a significant improvement was achieved with a *p* value of *p* = 0.005 when testing with an extended elbow, whilst the testing with a flexed elbow showed only a slight improvement with a *p* value of *p* = 0.372 not (Fig. 7).

Neither the measurement with the elbow extended (*p* = 0.735) nor the measurement with the elbow flexed (*p* = 0.677) showed significant differences between ‘Group A’ (Flexibar®) and ‘Group B’ (Flexibar® + XCO®).

#### Measuring the vibration sensation in 1/8 steps by using a 128 Hz tuning fork

**Fig. 8:**
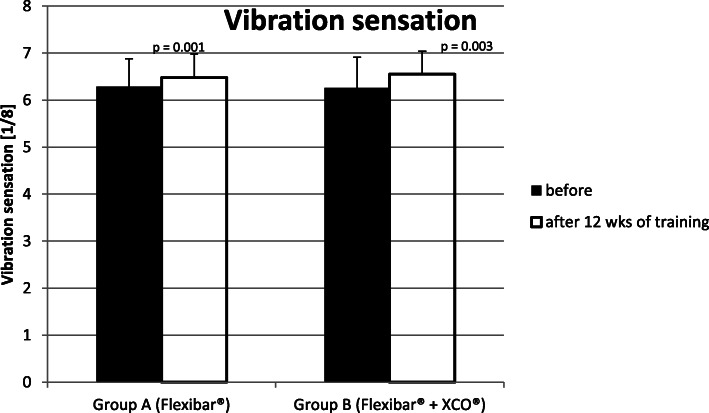
Development of the vibration sensation

In ‘Group A’ (Flexibar®), we could find an improvement of the vibration sensation for the symptomatic limbs on average from 6.3/8 ± 0.6/8 to 6.5/8 ± 0.5/8 and therefore about 3%. In ‘Group B’ (Flexibar® + XCO®), we could find an improvement for the symptomatic limbs on average from 6.3/8 ± 0.7/8 to 6.6/8 ± 0.5/8 and therefore about 5%. In both groups, a significant improvement of the vibration sensation was achieved with a *p* value of *p* = 0.001 in ‘Group A’ (Flexibar®) and *p* = 0.003 in ‘Group B’ (Flexibar® + XCO®) (Fig. 8), but without significant difference between the groups (*p* = 0.091).

#### Measuring the 2-point discrimination at the distal phalanx of each finger both radial and ulnar

**Fig. 9:**
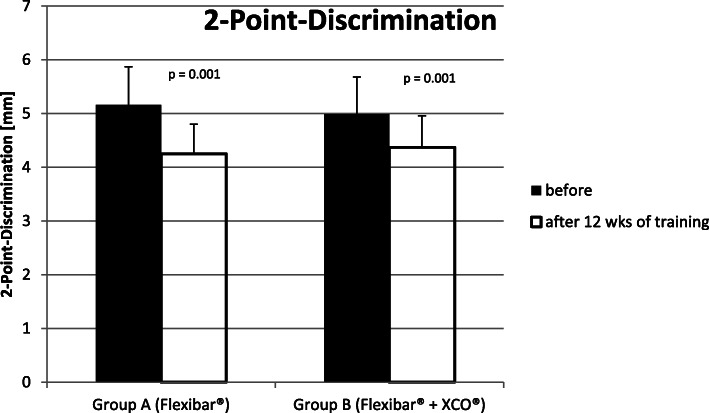
Development of the 2-point discrimination

In ‘Group A’ (Flexibar®), we could find an improvement of the 2-point discrimination for the symptomatic limbs on average from 5.2 mm ± 0.7 mm to 4.3 mm ± 0.6 mm and therefore about 18%. In ‘Group B’ (Flexibar® + XCO®), we could find an improvement of the 2-point discrimination for the symptomatic limbs on average from 5.0 mm ± 0.7 mm to 4.4 mm ± 0.6 mm and therefore about 12%. In conclusion, in both groups, we measured a significant improvement of the 2-point discrimination with a *p* value of *p* = 0.001 (Fig. 9), but there was no significant difference between the groups (*p* = 0.959).

## Discussion and conclusions

The primary purpose of any therapeutic effort for treating patients with lateral epicondylitis is the reduction of subjectively experienced pain by the patient which we put in the focus of our study as our primary goal parameter. In doing so, we were able to notice a continuous decline of the experienced pain intensity over the whole period of the study (see Fig. 4), so that both groups lastly achieved a significant reduction of the pain (34 % in ‘Group A’ (Flexibar®) and 40% in ‘Group B’ (Flexibar® + XCO®)). However, we could not determine a significant difference between both groups, so that we have to conclude that a more intensive training with different devices does not bring any additional benefit for the development of pain. In addition, we could verify a significant improvement of functionality of the upper limb in both groups, which we established by the DASH-Score (see Fig. 5). In both groups, a reduction of the point value was recorded by 56%, which corresponds to an even more remarkable improvement than the pain reduction. We could not find a significant difference between the both practice groups here either. As both parameters (VAS and DASH) are certainly mostly relevant for the contentment of the patients but despite everything have to be regarded as subjective parameters, we have added objectively measurable parameters to our analysis—the strength development, the vibration sensation and the 2-point discrimination. With regard to the measurement of strength development, it is hardly possible to objectively test the muscles that are primarily stressed by the training, so we used the grip strength according to Jamar as a measurable parameter. Since the muscles tested here are also stressed to a certain extent (firm grip of the equipment during the exercise), but are not the actual target muscle group of the treatment, another explanation for the improvement in the values could be assumed as follows: The most likely explanation is pain reduction and the thus facilitated exercise performance. In particular, we were able to observe significant improvements when performing the exercise with the elbow extended—which corresponds to the position that was initially already more painful. These values can therefore be interpreted as an improvement in functionality or discomfort rather than a pure increase in strength. In ‘Group A’ (Flexibar®), we found a significant improvement in both testings with the elbow extended and with the elbow flexed, whilst in ‘Group B’ (Flexibar® + XCO®), only the testing with the elbow extended showed a significant improvement and the testing with the elbow flexed only showed a slight improvement. The fact that ‘Group A’ (Flexibar®)—with the less intensive training—showed a better result here indicates that grip strength is not essential for the development of the first two goal parameters and that an increase in strength corresponding to the training intensity at least does not affect the tested muscle group. Furthermore, we also wanted to directly objectify the intended and expected effect on proprioceptive capabilities. For this purpose, we decided to test vibration sensation and 2-point discrimination in order to obtain measurable and comparable values. Again, we could prove a statistically significant improvement in both groups, both for the vibration sensation and also for the 2-point discrimination as an indication that the intervention was able to achieve the intended effect on the proprioception capability. Also here, a more intensive proprioceptive training with two different devices did not result in a bigger improvement of the proprioceptive capabilities. It should not be overlooked that this therapeutic option is bound to a sufficient compliance of the patients. Within our study, we asked the patients to keep a training diary that gave us the chance to evaluate the compliance. We could find that the training with the Flexibar® was rarely missed and sometimes even done more than required, whilst about 25% of the training sessions with the XCO® were missed. This is probably due to the fact that the flexibar exercises are easier to integrate into the daily routine of a working patient than jogging or walking exercises. The compliance as a very important factor of the success of this therapeutic options is also for certain one of the most relevant limitations. As a lot of patients hope for an immediate relief of their symptoms without any active support by themselves, a reduced compliance—beyond the setting of a clinical trial with a voluntary participation—is possible, whereby the therapeutic success could ultimately be reduced. Another limitation could be the correct execution of the exercise performance which is difficult to be controlled during autonomous training. Within the study, we gave exact exercise instructions to our patients at the beginning of the training period and also checked the exercise performance probatory at the beginning as well as at the end of the training period. Only 1-2 patients of each group showed an incorrect exercise performance, but we could also detect significant differences in the intensity of the execution; the influence of which on the outcome could not be measured. These differences may be attributed to the great heterogeneity of the collective of probands, but should also be seen in the public patient collective. Whether the collected results we have obtained can also be transferred to similar training devices of other manufacturers cannot be said with certainty, since we have not carried out a comparative study.

Overall, we were able to prove that the performance of a proprioceptive and ballistic training with the Flexibar® resulted in significant improvement of the subjectively experienced symptoms of the patients. However, a further benefit could not be shown by an additional training with the XCO®. Furthermore, we were able to measure other improvements with objective measurement parameters—in this case, the strength development, the vibration sensation and the 2-point discrimination—which demonstrated the effectiveness of this training.

Within comparative research in online databases, a large number of hits are found in search of clinical training studies for patients with lateral epicondylitis. Peterson et al. [14] demonstrated, for example, that an active training in patients with lateral epicondylitis can cause a more rapid relief of pain than a purely wait-and-see behaviour. In another clinical trial, Viswas et al. [15] found a superiority of a controlled exercise programme compared with a treatment with Cyriax physiotherapy. In addition, studies comparing a stretching with a consistent use of an orthosis [16] demonstrated an advantage of the active to the passive intervention. Various studies addressed the effect of an eccentric training of patients with lateral epicondylitis. At this, Söderberg et al. [17] could prove a significantly higher increase of a pain-free hand grip by combining an eccentric training with the use of an orthosis than the control group which received only the orthosis. Peterson et al. [18] finally compared two active training methods—eccentric versus concentric training—and found an advantage of the eccentric training over the concentric training for patients with lateral epicondylitis. However, all of the abovementioned studies have in common that no attention was paid to the reduced proprioception capability in the area of the elbow, which has been proven by Juul-Kristensen et al. [5]. However, examinations of other joints have already shown that proprioceptive training can have a positive effect on the functionality and reduction of symptoms in diseased joints. For comparison, other studies can be used which also detected a reduced proprioceptive capability in patients with symptomatic gonarthrosis [19]. Further studies demonstrated the effectiveness of proprioceptive training compared with a control group [20] and other results even show an advantage of a proprioceptive training compared with an isometric training [21]. Concerning the beneficial effects of a proprioceptive training for patients with lateral epicondylitis, only a few studies have been carried out yet. However, Tripp et al. [22] demonstrated in their study an improvement of the proprioceptive capabilities in the area of the elbow by performing a vibration training using vibrating dumbbells and varying frequencies. This trial used a collective of probands without a symptomativ lateral epicondylitis though. In the course of our study, we were able to prove that patients with lateral epicondylitis, which are more likely to have proprioceptive deficits as shown in other trials [5], active vibration training could cause an improvement of the proprioception itself as well as an improvement of grip strength, functionality and in particular pain reduction. In this setting, evaluated training devices can be used by the patients in the home environment, so that an independent training is possible without a commitment to a location or a fixed date and therefore the integration into the daily work routine of the primarily concerned working patient population is possible without difficulties.

One limitation of the study is that it was not possible to recruit a sufficient number of probands suffering permanent symptoms if there had been the chance that they could have been randomised to a group without treatment. Thus, a comparative assessment with the spontaneous outcome in our collective is not possible. Furthermore, we cannot make any statement about devices from other manufacturers, because we did not test them and it is not certain whether the results could be transferred in this way.

## Data Availability

The datasets generated and analysed during the current study are not publicly available due still contain data that have not yet been analysed but are available from the corresponding author on reasonable request.

## Abbreviations

DASH-Score: Score for the disabilities of the arm, shoulder and hand
VAS: Visual analogue scale
